# Use of non-Gaussian time-of-flight kernels for image reconstruction of Monte Carlo simulated data of ultra-fast PET scanners

**DOI:** 10.1186/s40658-020-00309-8

**Published:** 2020-06-19

**Authors:** Nikos Efthimiou, Kris Thielemans, Elise Emond, Chris Cawthorne, Stephen J. Archibald, Charalampos Tsoumpas

**Affiliations:** 1 PET Research Centre, Faculty of Health Sciences, University of Hull, Cottingham Rd, Hull, HU6 7RX UK; 2 Biomedical Imaging Science Department, School of Medicine, University of Leeds, Leeds, UK; 3 Department of Radiology, Perelman School of Medicine, University of Pennsylvania, 156B John Morgan Building, 3620 Hamilton Walk, Philadelphia, 19104-6055 PA USA; 4 Institute of Nuclear Medicine, University College London, London, UK; 5 Nuclear Medicine and Molecular Imaging, Department of Imaging and Pathology, KU Leuven, Leuven, Belgium; 6 Molecular Small Animal Imaging Centre, KU Leuven, Leuven, Belgium; 7 Invicro, Hammersmith Hospital, London, UK

**Keywords:** Monte Carlo, Positron emission tomography, Photon travel spread, Depth of interaction, Fast timing

## Abstract

**Introduction:**

Time-of-flight (TOF) positron emission tomography (PET) scanners can provide significant benefits by improving the noise properties of reconstructed images. In order to achieve this, the timing response of the scanner needs to be modelled as part of the reconstruction process. This is currently achieved using Gaussian TOF kernels. However, the timing measurements do not necessarily follow a Gaussian distribution. In ultra-fast timing resolutions, the depth of interaction of the *γ*-photon and the photon travel spread (PTS) in the crystal volume become increasingly significant factors for the timing performance. The PTS of a single photon can be approximated better by a truncated exponential distribution. Therefore, we computed the corresponding TOF kernel as a modified Laplace distribution for long crystals. The obtained (CTR) kernels could be more appropriate to model the joint probability of the two *in-coincidence* *γ*-photons. In this paper, we investigate the impact of using a CTR kernel vs. Gaussian kernels in TOF reconstruction using Monte Carlo generated data.

**Materials and methods:**

The geometry and physics of a PET scanner with two timing configurations, (a) idealised timing resolution, in which only the PTS contributed in the CTR, and (b) with a range of ultra-fast timings, were simulated. In order to assess the role of the crystal thickness, different crystal lengths were considered. The evaluation took place in terms of Kullback–Leibler (K-L) distance between the proposed model and the simulated timing response, contrast recovery (CRC) and spatial resolution. The reconstructions were performed using STIR image reconstruction toolbox.

**Results:**

Results for the idealised scanner showed that the CTR kernel was in excellent agreement with the simulated time differences. In terms of K-L distance outperformed the a fitted normal distribution for all tested crystal sizes. In the case of the ultra-fast configurations, a convolution kernel between the CTR and a Gaussian showed the best agreement with the simulated data below 40 ps timing resolution. In terms of CRC, the CTR kernel demonstrated improvements, with values that ranged up to 3.8*%* better CRC for the thickest crystal. In terms of spatial resolution, evaluated at the 60th iteration, the use of CTR kernel showed a modest improvement of the peek-to-valley ratios up to 1% for the 10-mm crystal, while for larger crystals, a clear trend was not observed. In addition, we showed that edge artefacts can appear in the reconstructed images when the timing kernel used for the reconstruction is not carefully optimised. Further iterations, can help improve the edge artefacts.

## Introduction

Time-of-flight (TOF) positron emission tomography (PET) takes advantage of the detection time difference between the two annihilation *γ*-photons to localise more precisely the position of the annihilation. This additional information makes the tomographic inverse problem less ill-posed [1].

The concept of TOF-PET was presented in the early 1980s [2, 3], when a reconstruction process using measurements from a scanner with TOF capabilities was formulated. The underlying radioactivity distribution was estimated using histograms, derived up to a scale to account for the speed of light [4–6]. However, early TOF-PET detectors suffered from overall poor timing performance (470 to 750 ps), low stopping power and light output which limited their spatial resolution and sensitivity.

Since the 2000s, substantial improvements of the timing resolution (from 600 to 310 ps) have been achieved thanks to major technological breakthroughs in the detector technology [7–13]. Most recently, a novel yet still currently premature technology capable of about 60-ps coincidence timing resolution (CTR) was presented [14].

The detection time differences for a given annihilation location and detector pair are usually considered to be normally distributed. Under this consideration, TOF reconstruction uses a Gaussian TOF kernel. However, Monte Carlo (MC) simulations suggest that this assumption is not accurate at ultra-high timing resolutions, e.g. below 50 ps [15–20].

Lately, experiments with Cherenkov photons from BGO crystals pointed out that the detection timing uncertainty could be more appropriately modelled with a mixture of two normal distributions [21, 22] or Lorentzian distribution [23]. Therefore, the Gaussian kernel might not be the most appropriate kernel under every configuration.

Several authors have presented experimental timing distributions which have a shape similar to the CTR kernel that we investigate in this study (possibly in convolution with a Gaussian) [24–26].

The effect of *over-* and *underestimation* of the width of the TOF kernel has been investigated at current timing resolutions [27]. However, to the best of our knowledge, the impact on image reconstruction of a non-Gaussian timing kernel for systems with very high timing resolution has not been studied yet. In a previous preliminary study [28], we used Laplacian TOF kernels. The Laplace distribution describes the difference of two independent exponential distributions; this is a reasonable approximation for the *γ*-photon absorption in long crystals.

In this paper, we investigate for the first time the use of TOF kernels for image reconstruction that model the crystal length. The application of this type of distributions is aimed at fitting data when the photon travel spread (PTS) in the crystals becomes significant or even dominant in the CTR.

As no systems with ultra-high timing resolution are available, in this paper, MC simulations were performed using the GATE simulation toolkit [29, 30] to model the geometry and physics of a PET system with very fast timing performance. The simulated data are then reconstructed with the proposed kernel and with a Gaussian kernel.

## Materials and methods

### Statistics of photon travel spread and coincidence timing

Briefly, the detection of *γ*-photon within scintillation detectors is a two-stage process. Firstly, the incident 511-keV photon is absorbed and optical photons are emitted within the scintillation crystal. Consecutively, a photo-detector converts these photons to electrical pulses [31].

When a narrow beam of 511-keV photons hits a scintillation crystal of thickness *L*, the original beam intensity (*I*_0_) is primarily attenuated due to photoelectric absorption or Compton scattering. In order to calculate the number of events absorbed in each depth layer inside the crystal, the following exponential model can be used:

1 $$ I(x) = I_{0}\exp(-\beta x)\,\text{,}\quad\text{for}\ 0\le x \le L   $$

where *β* approximates the material *absorption coefficient*.

**Fig. 1:**
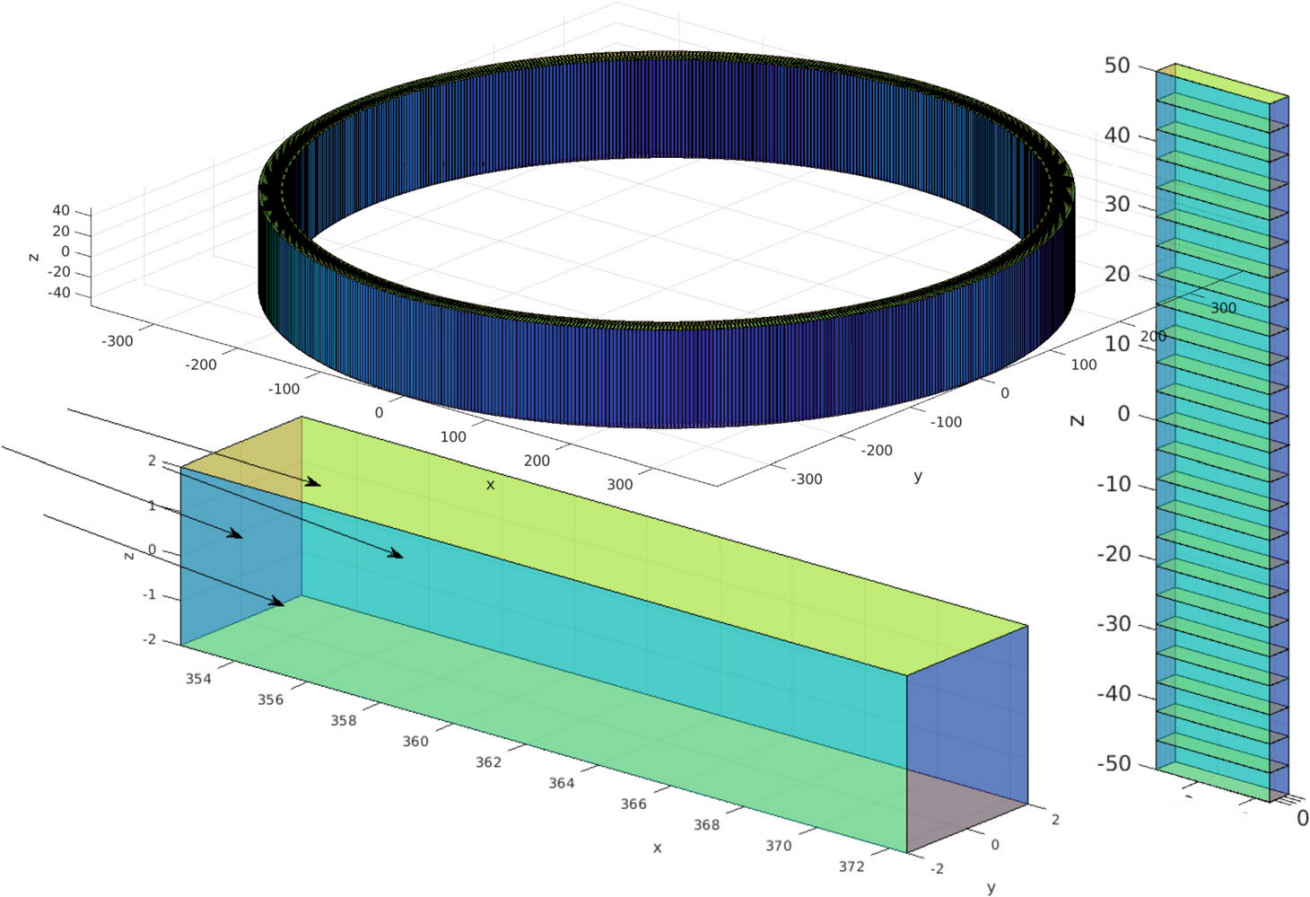
Illustration of the scanner’s geometry. The single crystals are grouped in modules with 24 crystals on the *z* axis, repeated 666 times around the FOV

Formula (1) can also be expressed in terms of time:

2 $$ I(t) = I_{0} \exp(- \lambda t)\, {,}\quad\text{for}\ 0\le t \le T   $$

where *λ*=*βc*, *c*≈0.2998 mm/ps is the speed of light and *T*=*L*/*c* (ps) is the maximum time duration of a *γ*-photon travelling perpendicularly to the entrance surface (Fig. 1).

The conditional probability density function (PDF) for events that are absorbed is obtained by normalising the above Eq. 2:

3 $$ g(t;\lambda) = \frac{\lambda\exp(- \lambda t)}{1 - \exp(-\lambda T)}\,\text{,}\quad\text{for}\ 0\le t \le T  $$

Let two photons *A* and *B* from a single annihilation event be independently detected. The continuous joint probability distribution is therefore *g*_*AB*_(*t*_*A*_,*t*_*B*_;*λ*)=*g*_*A*_(*t*_*A*_;*λ*)*g*_*B*_(*t*_*B*_;*λ*). Making the substitutions *d*=*t*_*A*_−*t*_*B*_, which is the time difference between the two photons, and *s*=*t*_*A*_+*t*_*B*_, we denote the joint distribution depending on *d* and *s* as:

4 $$ f_{DS}(d, s; \lambda) = g_{AB} (h_{1}(d,s), h_{2}(d,s); \lambda) \left| J \right|  $$

where *h* is the inverse transformation (*h*_1_(*d*,*s*)=(*s*+*d*)/2 and *h*_2_(*d*,*s*)=(*s*−*d*)/2) and *J* is the *Jacobian determinant* of *h*. Then, the CTR kernel can be obtained by integrating *f*_*DS*_ over *s*:

5 $$  f_{D}(d;\lambda) = \left(\lambda \sinh{\left(\lambda(T - \left|d \right|)\right)}\, \text{csch}\left(\frac{T\lambda}{2}\right)^{2}\right) \Big/ 4 \,\text{,}\quad\text{for} -T\le d \le T  $$

where csch is the hyperbolic cosecant function.

The corresponding cumulative distribution function (CDF) is given by:

6 $$ F_{D}(d;\lambda) = \frac{1 + \text{sgn}(d)}{2} - \text{sgn}(d) (\cosh{(\lambda(T - \left|d \right|))} - 1)\, \text{csch}\left(\frac{T\lambda}{2}\right)^{2} \Big/ 4  $$

where sgn is the sign function.

When *T*→*∞*, Eq. 5 becomes the Laplace distribution (*f*_*L*_) and *F*_*L*_ its CDF, given by:

7 $$\begin{array}{*{20}l} f_{L}(d; \lambda) &= \frac{\lambda}{2}\exp(-|d\lambda|) \end{array} $$

8 $$\begin{array}{*{20}l} F_{L}(d; \lambda) &= \frac{1}{2}\,\left(1 + \text{sgn}(d)\left(1-\exp(-\left|d\lambda\right|)\right)\right) \end{array} $$

In practice, other system effects will decrease the timing resolution of the system. This *non-idealised* case can be modelled by using a TOF kernel which is the convolution between the *f*_*D*_(*d*,*λ*) and a normal distribution *f*_*add*_(*d*,*σ*), with *σ* the standard deviation of the normal distribution. See the Appendix A.

### Scanner model

The geometry of a cylindrical PET scanner was simulated using the GATE simulation toolkit (v.8.1) [29, 32]. The scanner was comprised of 24 rings with 666 detectors each. No gaps between blocks were considered. The gap between the crystals was 0.2 mm. The inner ring radius was 424.5 mm (with field of view (FOV) radius 297 mm), and the total axial length was 110 mm. The crystals were made of Lu_2_Y_2_SiO_5_:Ce (LYSO) with density (*ρ*) equal to 7.105 g cm ^−3^. This crystal configuration provided realistic performance similar to the PreLude 420 by Saint Gobain [33]. The scanner’s geometry is illustrated at Fig. 1.

The surface of each scintillation crystal was 4×4 mm^2^. Different crystal lengths were used, which are defined in each section. The energy resolution was set to 11.4*%*, and the applied energy window was 435–650 keV. The coincidence timing window was set to 4.1 ns. Each crystal measurement was read out individually without summing up the energy from neighbouring crystals. The minimum allowed radial detector difference (rsector difference in GATE) was 83 detectors. The emstandardemstandard_opt3 physics list was used.

#### System’s timing resolution

Although various methods for the evaluation of the timing resolution of a PET scanner have been proposed [34–37], in this study, a simpler approach was considered.

To investigate the effect of the PTS on the CTR, a thin (0.05 mm radius) *back-to-back* *γ*-photon rod source was simulated until approximately 70×10^6^ total events were recorded.

In GATE, the “macro” command setTimeResolution applies an additional normal blurring to the detection time for each detector, i.e. following the original detection time defined as the time of occurrence of the photoelectric effect (PE) in the crystal.

Using the aforementioned “macro” command, two detector configurations were considered:

1. First case, the additional detector time resolution was set to 0 ps which simulated a detector with *idealised* timing properties. In this case, the CTR kernel was tested for three different crystal thicknesses, namely 10, 20 and 40 mm. For comparison, a normal distribution was fitted to the simulated timing responses using maximum likelihood estimate (MLE) (via the Distribution Fitter App in Mathworks Matlab). With regard to the data boundaries, two cases were considered. In the first case, data boundaries were placed such that we obtained the minimum Kullback–Leibler (K-L) distance (optimum kernel) between the simulated distribution and the timing kernel (*f*_*N*_). On the other hand, on the second fitting ($f^{\prime }_{N}$), no data boundaries were placed.
2. Second case, a range of values were set to the setTimeResolution, simulating a *non-ideal* detector. The crystal size was fixed to 20 mm, as this is the one of most common thicknesses for ^176^Lu-based crystals [38–40]. Seven additional detector timing resolutions were considered FWHM_add_ = 0, 5, 10, 20, 40, 60, 80 and 100 ps. This additional timestamp smearing represents other factors affecting the timing spread, such as fluctuation in the detection of optical photons, pulse integration and electronic noise. According to the central limit theorem, the additional timing uncertainty due to all these effects can be described by the normal distribution. The two kernels (i.e. the additional timing kernel and the CTR) are then convolved. As such, the shape of the final kernel depends on both kernels.

In addition, the histograms of the timing differences corresponding to the *non-idealised* scanners were compared with the convolution kernel given in the Appendix A (Eq. 11).

### Image reconstruction

#### Average depth of interaction

STIR takes into account an average depth-of-interaction (DOI) effect in the crystal for the calculation of the line of response (LOR)’s position. In order to find a good approximation for the average DOI, the detected *γ*-photons were binned into histograms based on the depth where they were absorbed. Then, the mean absorption depth was found by summing the bin values until the mean value was found.

The average DOI values were found to be 3.6, 5.8 and 7.4 mm for the 10-, 20- and 40-mm crystals, respectively.

#### Calculation of the TOF projection matrix

The TOF kernel, as implemented in STIR, is applied on top of the non-TOF LOR (*p*_*ij*_) as [41]:

9 $$  \begin{aligned} p_{it;j} &= p_{ij} K_{it;j},\\ K_{it;j} &= \text{cdf}(k_{t+1} - v'_{cj}) - \text{cdf}(k_{t} - v'_{cj}) \end{aligned}  $$

where *K*_*it*;*j*_ is the time response for the *t*th TOF position of the *i*th bin and *j*th image element, cdf is the CDF corresponding to the timing kernel used, [*k*_*t*_,*k*_*t*+1_) is the timing interval for the *t*th TOF bin and $v^{\prime }_{cj}$ is the projection of the voxel’s centre on the TOF line.

#### Reconstruction algorithm

STIR [42, 43] supports a wide range of algorithms for the determination of the maximum likelihood estimate (MLE), including ordered subset expectation maximization (OSEM), median root prior (MRP) and quadratic prior (QP) Bayesian one step late methods [44, 45], and the ordered subset separable paraboloidal surrogates algorithm [46].

In this paper, listmode (LM)-maximum likelihood-expectation maximisation (MLEM) was used [47, 48] as it is the simplest option, and is guaranteed to converge (even slowly) to a solution. The TOF version of LM-MLEM in the STIR library was previously presented with simulated data [41] and recently validated using measured PET data [49]. The size of the TOF bins was 1 ps (numbering 4101 in total). No TOF mashing, view mashing or axial compression was used for the data.

The voxel size of the reconstructed images was 1×1×2.08 mm^3^. In order to reduce the reconstruction duration, the number of voxels was adjusted to fit the size of the phantom in each case. No post-reconstruction smoothing filters were applied to the images.

Attenuation correction factors were calculated with an analytical simulation, of the phantom, having the appropriate linear attenuation values for 511-keV *γ*-photons, as found in NIST [50]. Normalisation factors were not used. The scattered and random events were omitted from the reconstructions; all datasets had 40×10^6^ true events.

The iterative process was performed for up to 105 iterations for the contrast recovery coefficient (CRC) and 150 iterations for the spatial resolution. However, all results are discussed for the 60th iteration, as it ensures that region of interest (ROI) values have almost converged, without introducing noise amplification and reduction in signal to noise ratio [51].

Further, expansion of the software allowed us to parallelise the TOF LM-MLEM reconstruction using OPEN-MP. STIR supported the options for OPEN-MP and MPI for reconstruction of sinograms only. The new code reduced the amount of time needed for a single iteration 10× running with 25 threads on 28 processor on the University’s cluster.

### Simulated phantoms

#### NEMA image quality: contrast recovery coefficients

A NEMA image quality phantom [52] was designed and simulated for all scanner geometries under consideration.

The CRC of a hot sphere with inner diameter *d* was calculated as:

10 $$ \textrm{CRC}_{r} = \left. \left(\frac{\mu_{H,d}}{\mu_{B,d}} - 1\right) \middle/ (\alpha - 1) \right.  $$

where *α*=4.5 is the actual contrast ratio of the sphere, *μ*_*H*,*d*_ is the mean value of the ROI and *μ*_*B*,*d*_ is the mean value of the background in the reconstructed images. The inner diameters of the hot spheres were 10,13,17 and 22 mm. In order to reduce the statistical error, the simulations were repeated 7 times; *μ*_*H*,*d*_ and *μ*_*B*,*d*_ values were averaged over all datasets.

#### Spatial resolution

In order to evaluate the effect of the different kernels on the spatial resolution, a computational Derenzo-*style* phantom was simulated. The phantom material was set to plastic (as defined in the GATE materials database) with a 5-cm radius and a 7-cm height. The hot rods were subdivided into six sections, with diameters of (A) 7.0 mm, (B) 5.0 mm, (C) 4.0 mm, (D) 3.5 mm, (E) 3.0 mm and (F) 2.5 mm (the letters denote the name-ID of each section). The separation distance between the rods was set to the double of their diameter [53]. In total, 40×10^6^ true events were used to reconstruct the images.

As previously discussed [54], the assessment of the actual image resolution with statistical image reconstruction is not trivial as spatial resolution depends on the iteration number and activity distribution. In order to limit the effect of the non-negativity constraint, a high activity background source was used.

Furthermore, in order to evaluate whether the reconstructed sources contain edge artefacts, the ratio between the pixel value on the centre of gravity (COG) of the source and the average ROI value was recorded for the sources of the largest section.

## Results

### Comparison between the CTR kernel and simulated data

**Fig. 2:**
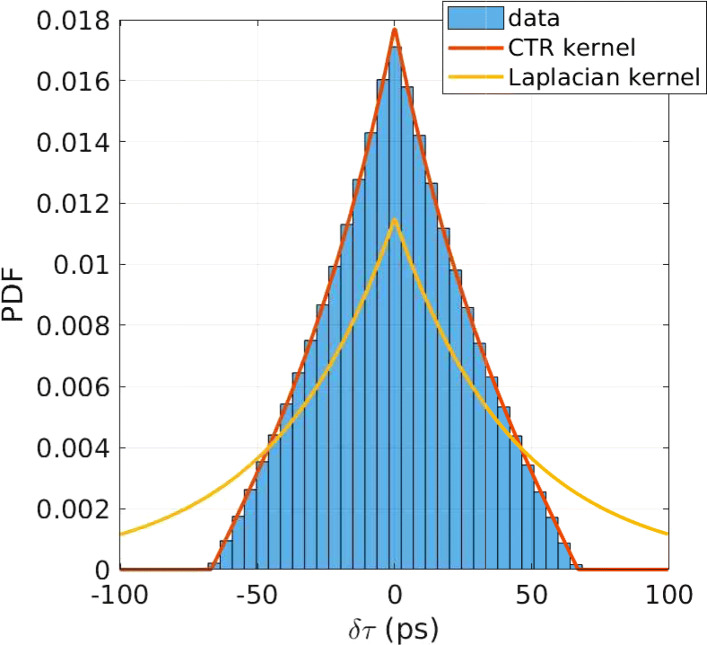
The CTR kernel (*f*_*D*_) compared to the histogram of simulated time differences for a crystal thickness of 20 mm. For comparison, the Laplace (*f*_*L*_) with the same lambda is shown, i.e. corresponding to an infinitely thick crystal

Figure 2 shows a comparison between the CTR kernel (*f*_*D*_) and the Laplace (*f*_*L*_) with *idealised* simulated time differences for a crystal thickness of 20 mm. As shown, the *f*_*D*_ is in excellent agreement with the simulated data, while the tails of the Laplacian kernel extend to infinity.

### Timing response for different crystal lengths in the idealised case

**Fig. 3:**
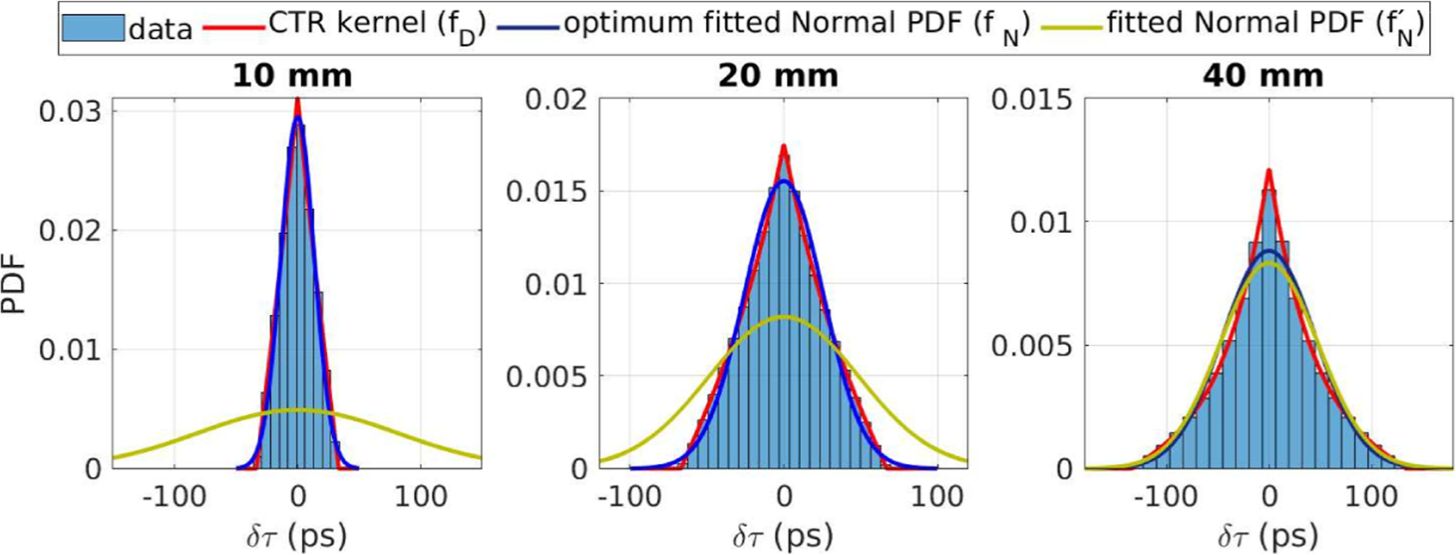
Fits of a normal distribution and an analytically calculated CTR kernel, compared to the idealised simulated timing responses for crystal lengths equal to 10, 20 and 40 mm

The simulated timing PDFs for three different crystal lengths with the idealised timing response in comparison with the corresponding CTR kernel, an unconstrained fitted normal distribution ($f^{\prime }_{N}$) and a fitted normal distribution with boundaries which minimise the K-L distance between the kernel and the data (*f*_*N*_), are shown in Fig. 3. The system’s timing resolution (FWHM_T_) of the $f^{\prime }_{N}$ was found to be 192.2, 118.0 and 111.1 ps, for the 10-, 20- and 40-mm crystal size, respectively.

The corresponding values for the *f*_*N*_ were 31.2 ps, 60.5 ps and 106.6 ps. This difference is attributed only to the effect of the crystal’s length. It should be noted that the difference between the two Gaussian kernels is significant for the smaller crystals.

The K-L distances between the optimum normal (*f*_*N*_) and measured PDF were found to be equal to 0.020, 0.027 and 0.059 for the 10-, 20- and 40-mm crystal, respectively. For the analytically calculated *f*_*D*_, the corresponding values were all below 8×10^−4^.

### Non-idealised timing response

**Fig. 4:**
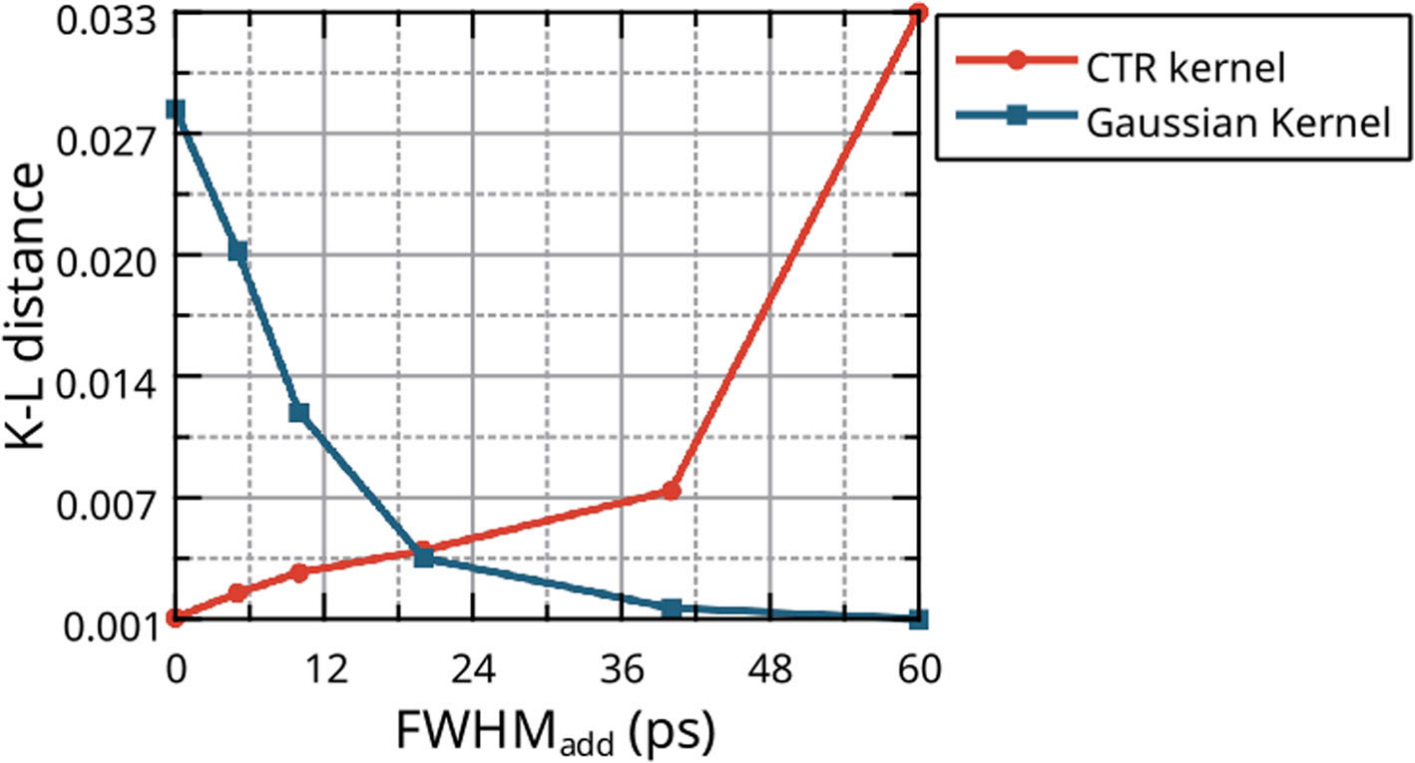
Absolute K-L distances between the non-idealised simulated data of the 20-mm crystal, a fitted normal distribution (*f*_*N*_) and the CTR kernel

When considering a scanner with *non-idealised* timing response, the CTR kernel *f*_*D*_ might no longer be appropriate. The absolute K-L distances of the optimised fitted normal distribution (*f*_*N*_) and the CTR kernel (*f*_*D*_) with the measured histogram for a 20-mm crystal can be found in Fig. 4.

**Fig. 5:**
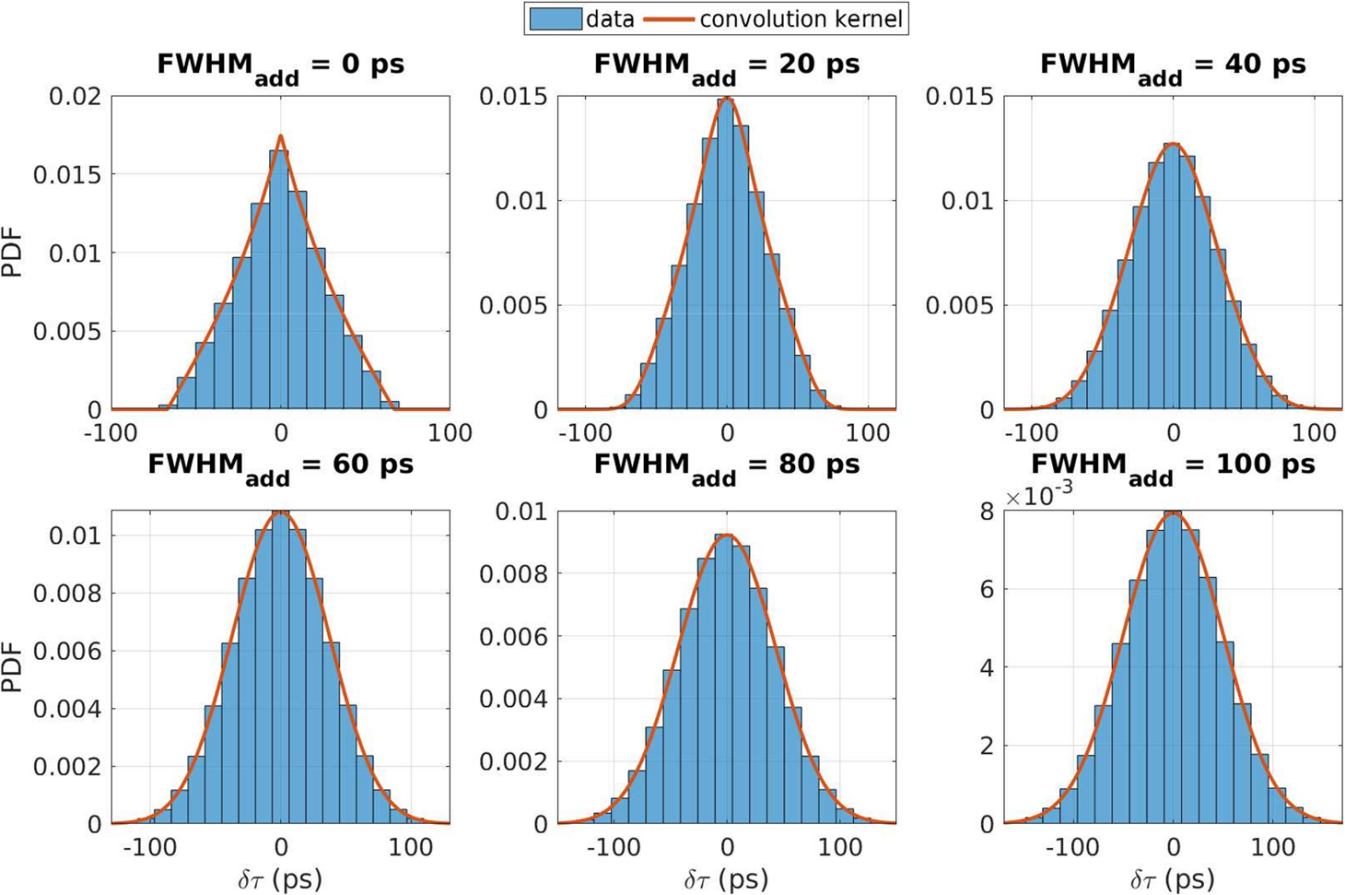
Histograms of simulated time differences, with additional normal timing blurring with various FWHMs ranging from 0 to 100 ps. In addition, the corresponding convolution kernels generated using the CTR kernel for the 20-mm LYSO crystal and the respective additional normal

As one may see, for the 20-mm LYSO crystal below FWHM_add_ = 20 ps, the CTR kernel is in better agreement with the simulated data than the fitted normal distribution. In addition, when the additional timing blur is below 60 ps, the timing response should not be considered as following a pure normal distribution. In this case, a convolution (Appendix A) between the two distributions should be considered as shown in Fig. 5.

### Image reconstruction

#### Contrast recovery coefficient

**Fig. 6:**
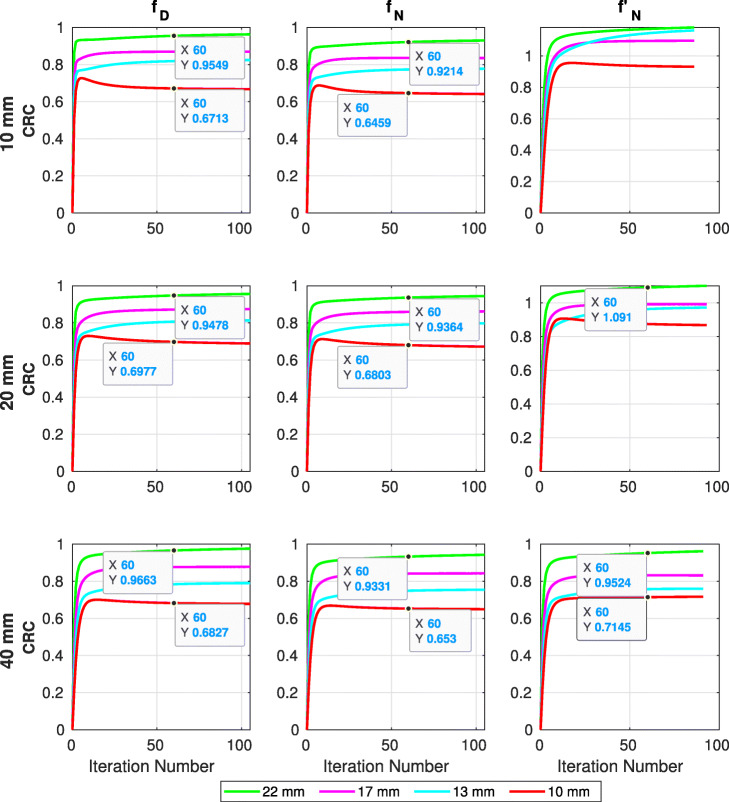
CRCs for all spheres and crystal sizes with an idealised scanner, using different kernels. CTR (*f*_*D*_), Gaussian (with data boundaries) (*f*_*N*_) and Gaussian (no data boundaries) ($f^{\prime }_{N}$)

Overall, the performance of both *f*_*D*_ and *f*_*N*_ was very good, as expected by such fast timing resolutions. Contrast recovery reached close to 1 for the larger sphere and about 0.7 for the smallest 10-mm sphere (Fig. 6). The results show that the better the agreement of the kernel with the data, the better contrast is recovered.

The CTR kernel (*f*_*D*_) for all crystal sizes performed better than the Gaussian kernels. Their performance gap was the smallest for the 20-mm crystal, which was the crystal that the Gaussian kernel provided its best values. With respect to the crystal size, we saw that the size of the crystal had a positive impact.

Use of the un-optimised normal kernel ($f^{\prime }_{N}$) led to poor CRC accuracy as in many cases the values were artificially increased above 1.0. This overshoot was more intense for the smaller crystals, where the agreement of the kernel with the data was at its lowest. However, on the 40 crystal, the performance was comparable to that of the optimised kernel.

#### Spatial resolution (peak-to-valley ratio)

**Fig. 7:**
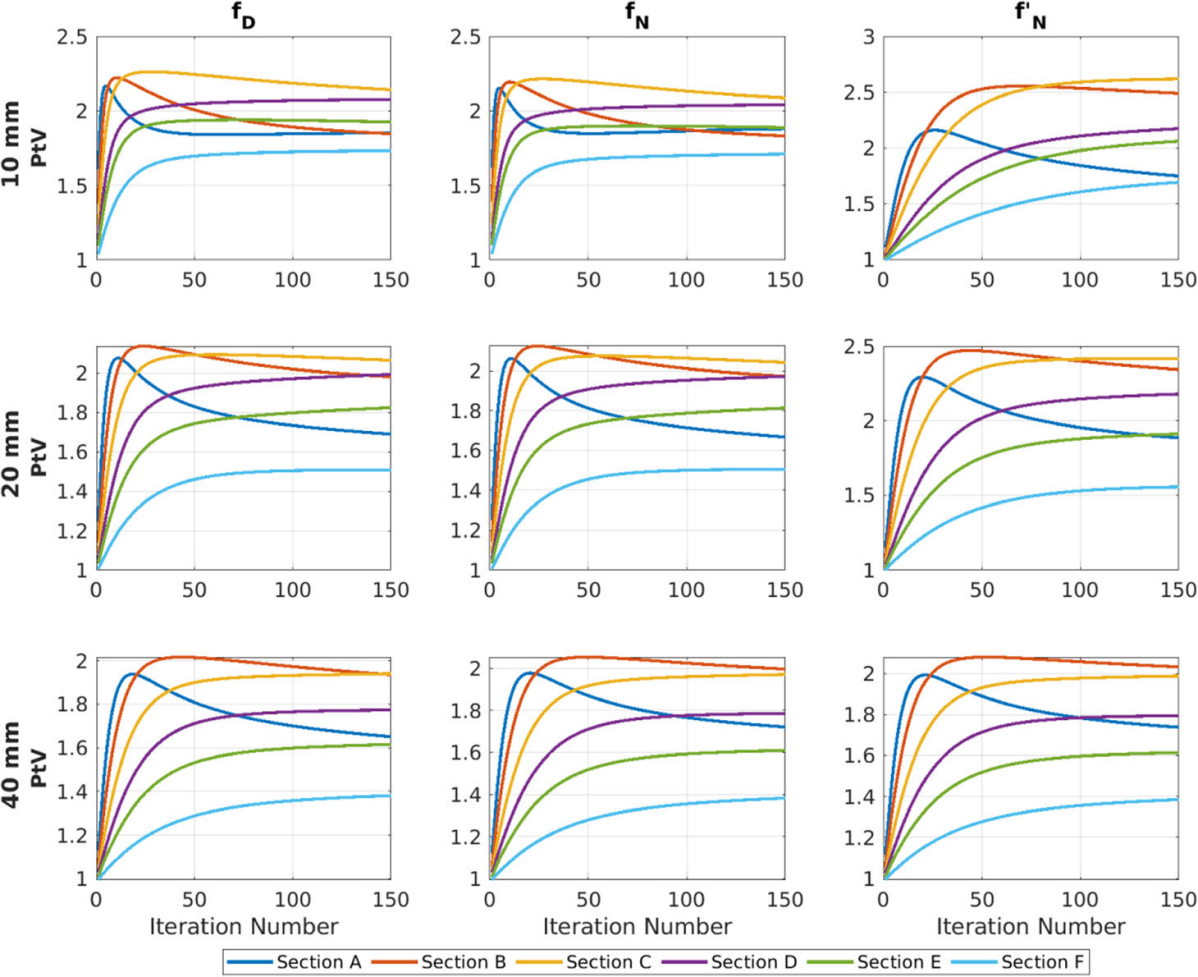
Peak-to-valley ratios of all combinations of crystals with an idealised scanner as function of the iteration number. The six rod section had diameters of (A) 7.0 mm, (B) 5.0 mm, (C) 4.0 mm, (D) 3.5 mm, (E) 3.0 mm and (F) 2.5 mm, for the CTR (*f*_*D*_), Gaussian with data boundaries (*f*_*N*_) and without data boundaries ($f^{\prime }_{N}$) kernels

The PtV ratio for a wide range of iterations is presented on Fig. 7. Overall, the CTR kernel performs better than the other kernels, for thinner crystals. In addition, the data shown that for smaller crystals, the spatial resolution converges faster.

The ratio for section C (4.0 mm) and the 10-mm crystal, evaluated at the 60th iteration, was 2.203 for the CTR kernel and 2.151 for *f*_*N*_. An improvement of 1%.

However, for the 40-mm crystal, not all sections followed similar trends. Sections A, B and C provided higher peak-to-valley (PtV) ratios with the Gaussian kernel, while the smaller sections D, E and F with the CTR.

**Fig. 8:**
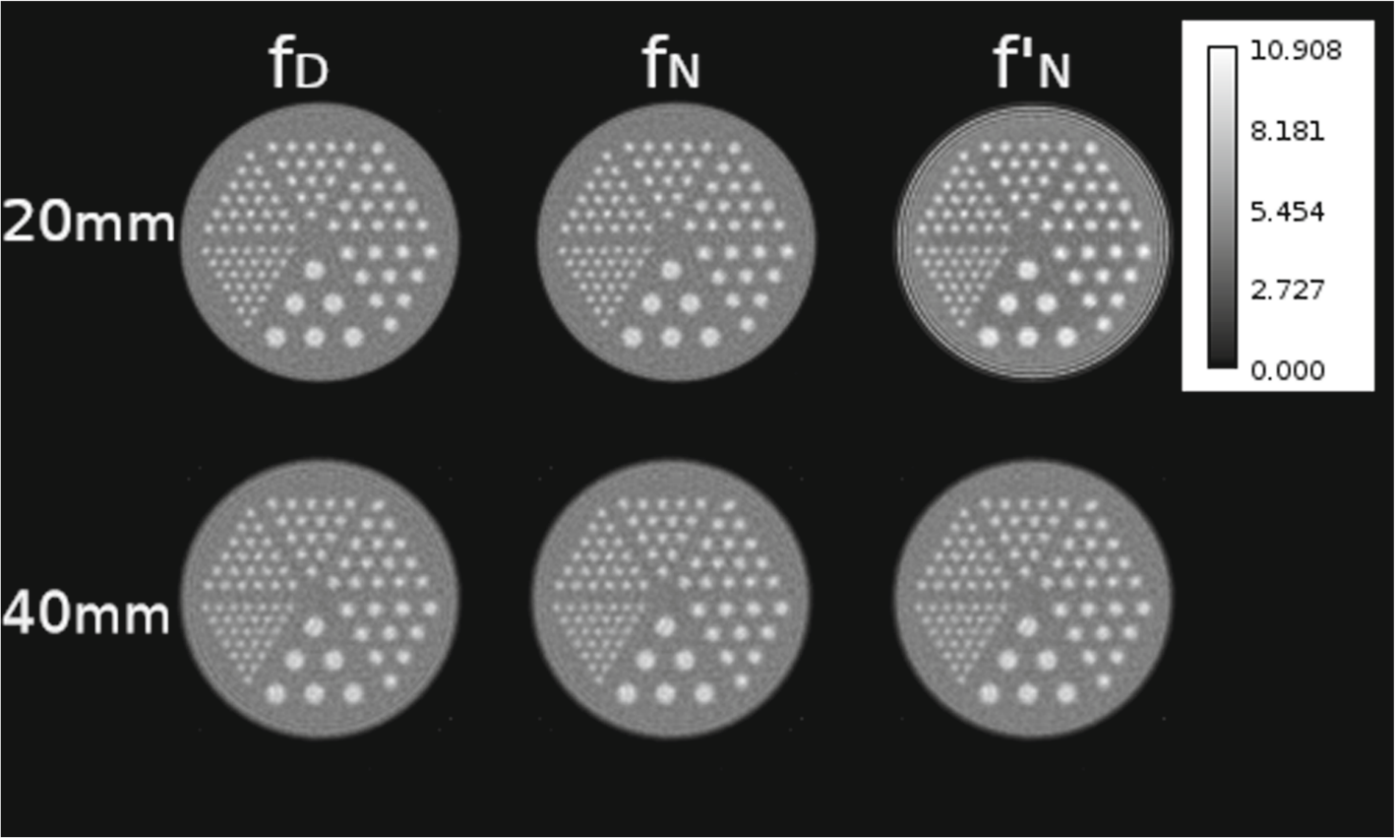
Reconstructed images with TOF LM-MLEM on the 60th iteration. The images are the sum over all axial slices. The images have been scaled to the global max value

As the iterative process progressed, growing presence of edge artefacts affecting the spatial resolution in the reconstructed images was observed. These artefacts depended on the size of the source, crystal size, type of kernel and iteration number. They looked like an overshoot or ringing after sharp transitions of intensity in the images (Fig. 8).

Their appearance was spotted at earlier iterations for the larger sources (e.g. of section A), hence the quick reduction of PtV observed in Fig. 7, and smaller crystals. Sources of the section D and smaller did not exhibit this artefact up to the 150th iteration.

For instance, at Fig. 8 the 20-mm crystal, 60th iteration, the sources of the section A present a ring*-like* shape. The reduction in the PtV ratios (Fig. 7) strongly correlates with the presence of this artefact.

**Fig. 9:**
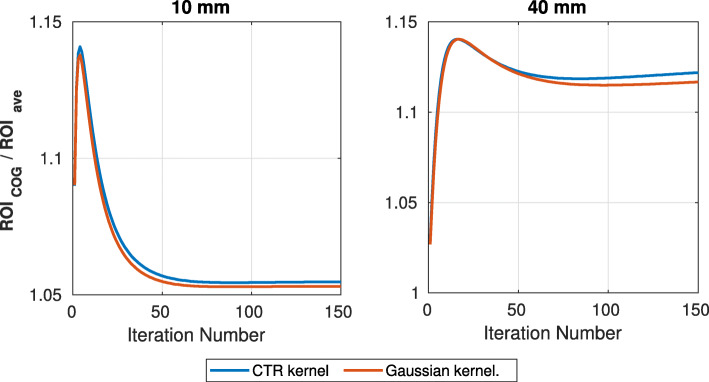
Ratio between the value of the voxel on the centre of gravity of the ROI over the average value of the ROI. Reduction on the ratio value happens due to edge artefacts

Figure 9 shows the ratio between the intensity of the voxel on the ROI’s COG and its average value for section A, crystals 10 and 40 mm. This ratio provides an indication of the magnitude of the edge artefact.

As it can be seen, the ratios peak at early iterations (4 and 16, respectively), followed by a drop (due to the edge artefact). As the results show, the effect on the 10-mm crystal is more intense. However, there are indications that further iterations improve the ratios. In addition, it is shown that the CTR kernel recovers the ratio faster than the Gaussian. This further demonstrates the importance of accurate timing modelling.

Moreover, on the third column of Figs. 7 and 8, the results of the kernel ($f^{\prime }_{N}$) are shown. As it can be seen for the case of the 20-mm crystal, severe ring artefacts are present on the background activity, which are not apparent for the 40-mm crystal. The poor fitting of the $f^{\prime }_{N}$ kernel with the simulated time differences, for the 20-mm crystal, is the driving factor for these rings.

## Discussion

In this paper, we compared images reconstructed using TOF kernels calculated using different methodologies. In the comparison, we included Gaussian kernels, which are the standard kernels being used currently, and a modified Laplacian kernel which accurately models the effect of the PTS in the crystal (and thereby timing differences due to DOI) on the system’s timing response.

The data used in this manuscript were generated using GATE MC simulations of *idealised* and ultra-fast PET scanners based on LYSO detectors. In particular, we focused on systems where the CTR is very close to the ideal case, where the only time-degrading effects are due to the uncertainty in location of the *γ*-photon absorption.

As was shown, when the size of the crystal is the only parameter affecting the timing performance of the detector (*idealised* scanner), the PTS distribution shapes the timing response. In good approximation, the PTS depends on the exponential absorption of the *γ*-photons in two crystals. Therefore, the timing kernel can be approximated as the convolution between two truncated exponential functions. If the crystals were long enough to absorb 100% of the annihilation photons, this function would be simplified to the Laplace distribution.

In addition, our results illustrate that below FWHM_add_ = 40 ps, the PTS gradually becomes a dominant factor in shaping the distribution of the timing measurements. As such, in this range, the Gaussian function does not appear to be a good descriptor for the timing differences, but the convolution kernel which considers the CTR is preferred.

In order to investigate the effect of the proposed, accurate, timing modelling in the image reconstruction, contrast recovery and spatial resolution were used as figures of merit. The evaluation included two computational phantoms: the NEMA IQ and a Derenzo-*style* phantom.

In addition, for comparison, two Gaussian kernels were considered: the optimised (*f*_*N*_), for which data boundaries were placed when fitting, and the un-optimised (*f*_*N*_), without data boundaries (*f*_*N*_).

The reconstructed images of the simulated NEMA IQ phantom showed in the case of the *idealised* scanner that in terms of CRC, the CTR kernel performed up to 3.5*%* better for the 22-mm sphere and 3.8*%* for the 10-mm sphere, compared to the optimised Gaussian kernel (*f*_*N*_). This can be explained in Fig. 5 as the CTR kernel is the only one able to capture accurately the peak of the simulated timing differences.

In addition, we demonstrated that when the agreement between the kernel with the data is poor ($f^{\prime }_{N}$ kernel with 10- and 20-mm crystals), there is a significant overshoot in CRC, which can reach up to 20%, in our case. The magnitude of this overshoot depends on the size of the crystal size.

The spatial resolution was evaluated using the PtV ratios, at the 60th iteration. It was shown that the CTR kernel offered a relatively small advantage of about 1% for the thinnest 10-mm crystal, while for the 20-mm crystal, the advantage of the CTR kernel was significantly reduced. Finally, the 40 mm had mixed results as the Gaussian kernel performed better for the largest sections and the CTR for the smaller.

However, it should be noted that the majority of ^176^Lu-based detectors have lengths from 12 to 20 mm [38–40, 55].

Evaluation of the PtV for the not optimised Gaussian kernel ($f^{\prime }_{N}$) was not possible, due to the overshoot in contrast values, as demonstrated in the earlier paragraph.

Edge artefacts were observed in the images of the Derenzo-*style* phantom. In the case of point spread function (PSF) modelling, edge artefacts are present due to the ill-conditioned nature of the problem [56] and depend on the size of the source and the iteration number [57]. Similar behaviour was observed here for the TOF reconstructions. However, in our case, we applied only a basic PSF modelling. We note that these types of artefacts are not apparent in the non-TOF reconstructed images (not shown here).

A potential explanation of the edge artefacts is on the fact that there are some discrepancies between the TOF kernel and the MC data. Good agreement of the timing kernels with the simulation is not sufficient for very fast timing detectors, because small spatial displacements can affect positioning the timing kernel accurately. For example, spatial displacements can be caused by approximating the scanner as a continuous cylinder rather than a polygon-cylinder with gaps [58], due to consequent differences in the DOI and parallax effect.

This hypothesis is supported by the fact that the artefacts were reduced when a more accurate CTR kernel was used. In addition, when a kernel of poor agreement ($f^{\prime }_{N}$) (crystals 10 and 20 mm) with the data was used, intense ring artefacts appeared in the background activity of phantom and these rings disappeared when $f^{\prime }_{N}$ fitted better the data (40-mm crystal). This may also indicate that at high timing resolution, image quality becomes more dependent on TOF kernel accuracy. However, this needs further investigation. It should be pointed out that for ultra-fast timing resolution, and in particular when the size of the TOF kernel becomes comparable to the voxel size, the detection probability is no longer separable between the spatial and timing response and Eq. 9. The simplest strategy to address this would be to use smaller voxels, but this would lead to increased computational requirements and an increase of noise. Thus, regularised reconstruction would therefore become essential.

In addition, currently, STIR does not model sufficiently the detector’s response [59] or other effects such as inter-crystal scattering.

The major limitation of the current study is that the simulations for optical photons inside the crystals [60–63] and their processing (i.e. pulse integration) [64] were not considered due to their substantially computational requirements. In addition, the details of the optical photon transport and further processing will depend on the actual detector design. We have presented results where such effects are modelled via an additional normal distribution blurring. This would have to be tested for a particular detector.

Indeed, at the time of writing, it is not known whether future detectors will exhibit the proposed timing distribution. However, as new technological advances progress towards the 50-ps range, accuracy in timing detector modelling will become essential [65].

## Conclusion

The use of TOF kernels, which take into account the effect of the PTS in the crystal, matches the simulated timing differences better than the traditional Gaussian kernel, when ultra-fast detectors are considered. In the presence of additional timing spread, convolution between the proposed CTR kernel and a Gaussian provided a very accurate model for the timing measurements.

However, even if the use of the CTR is in better agreement with the simulated data, the observed improvements in the reconstructed images in terms of contrast recovery and spatial resolution are modest on the range of 1 to 3.5*%*.

## Appendix A: Convolution formula

The following formula calculates the convolution kernels between the *f*_*D*_(*d*;*λ*) for the range [−*T*,*T*] and *f*_add_(*d*,*σ*):

11 $$ f_{conv}(d, \lambda, T, \sigma) = \frac{ A\Big((1+\exp{(2T\lambda)}) B -C + \exp{(2T\lambda)D} + H + J\Big) } {2(1+ \exp{(2T\lambda)})(-1 + \cosh{(T\lambda)})\sqrt{2\pi} }   $$

where

12 $$\begin{array}{*{20}l} A &= \exp{\left(0.5\lambda\left(-2d + \lambda\sigma^{2}\right)\right)} \sqrt{0.5\pi}\lambda\cosh{(T\lambda)} \end{array} $$

13 $$\begin{array}{*{20}l} B &= \text{erf}\left(\frac{d-\lambda\sigma^{2}}{\sqrt{2}\sigma}\right) \end{array} $$

14 $$\begin{array}{*{20}l} C &= \text{erf}\left(\frac{T + d - \lambda\sigma^{2}}{\sqrt{2}\sigma}\right) \end{array} $$

15 $$\begin{array}{*{20}l} D &= \text{erf}\left(\frac{T - d + \lambda\sigma^{2}}{\sqrt{2}\sigma}\right) \end{array} $$

16 $$\begin{array}{*{20}l} E &= \text{erf}\left(\frac{d + \lambda\sigma^{2}}{\sqrt{2}\sigma}\right) \end{array} $$

17 $$\begin{array}{*{20}l} F &= \text{erf}\left(\frac{-T + d + \lambda\sigma^{2}}{\sqrt{2}\sigma}\right) \end{array} $$

18 $$\begin{array}{*{20}l} G &= \text{erf}\left(\frac{T + d + \lambda\sigma^{2}}{\sqrt{2}\sigma}\right) \end{array} $$

19 $$\begin{array}{*{20}l} H &= \exp{(2d\lambda)}\left(- E + F\right) \end{array} $$

20 $$\begin{array}{*{20}l} J &= \exp{(2(T+d)\lambda)}\left(-E + G\right) \end{array} $$

## Data Availability

The datasets used and analysed during the current study are available from the corresponding author on reasonable request.
