# Correction to: Use of non-Gaussian time-of-flight kernels for image reconstruction of Monte Carlo simulated data of ultra-fast PET scanners

**DOI:** 10.1186/s40658-022-00441-7

**Published:** 2022-02-24

**Authors:** Nikos Efthimiou, Kris Thielemans, Elise Emond, Chris Cawthorne, Stephen J. Archibald, Charalampos Tsoumpas

**Affiliations:** 1 PET Research Centre, Faculty of Health Sciences, University of Hull, Cottingham Rd, Hull, HU6 7RX UK; 2 Biomedical Imaging Science Department, School of Medicine, University of Leeds, Leeds, UK; 3 Department of Radiology, Perelman School of Medicine, University of Pennsylvania, 156B John Morgan Building, 3620 Hamilton Walk, Philadelphia, PA 19104-6055 USA; 4 Institute of Nuclear Medicine, University College London, London, UK; 5 Nuclear Medicine and Molecular Imaging, Department of Imaging and Pathology, KU Leuven, Leuven, Belgium; 6 Molecular Small Animal Imaging Centre, KU Leuven, Leuven, Belgium; 7 Invicro, Hammersmith Hospital, London, UK

## Correction to: EJNMMI Phys (2020) 7:42 https://doi.org/10.1186/s40658-020-00309-8

Following publication of the original article [1], two typographical errors were found by the authors in formulas 6 of the main text and 19 in the appendix. The original and correct versions of the equations are given below:

Original formula 6: $$F_{D}(d;\lambda) = \left(\frac{1 - \text{sgn}(d)}{2} - \text{sgn}(d) (\cosh{(\lambda(T - \left|d \right|))} - 1)\, \text{csch}\left(\frac{T\lambda}{2}\right)^{2}\right) \Big/ 4$$.

Correct formula 6: $$F_{D}(d;\lambda) = \frac{1 + \text{sgn}(d)}{2} - \text{sgn}(d) (\cosh{(\lambda(T - \left|d \right|))} - 1)\, \text{csch}\left(\frac{T\lambda}{2}\right)^{2} \Big/ 4$$.

Original formula 19: $$H=exp(2d\lambda)(E+F)$$.

Correct formula 19: $$H=exp(2d\lambda)(-E+F)$$.

The original article [1] has been corrected.
